# Differential Expression Profile of NLRs and AIM2 in Glioma and Implications for NLRP12 in Glioblastoma

**DOI:** 10.1038/s41598-019-44854-4

**Published:** 2019-06-11

**Authors:** Nidhi Sharma, Shivanjali Saxena, Ishan Agrawal, Shalini Singh, Varsha Srinivasan, S. Arvind, Sridhar Epari, Sushmita Paul, Sushmita Jha

**Affiliations:** 1 Department of Bioscience and Bioengineering, Indian Institute of Technology Jodhpur, Jodhpur, India; 2 Department of Pathology, Tata Memorial Hospital, Mumbai, Maharashtra India

**Keywords:** NOD-like receptors, CNS cancer

## Abstract

Gliomas are the most prevalent primary brain tumors with immense clinical heterogeneity, poor prognosis and survival. The nucleotide-binding domain, and leucine-rich repeat containing receptors (NLRs) and absent-in-melanoma 2 (AIM2) are innate immune receptors crucial for initiation and progression of several cancers. There is a dearth of reports linking NLRs and AIM2 to glioma pathology. NLRs are expressed by cells of innate immunity, including monocytes, macrophages, dendritic cells, endothelial cells, and neutrophils, as well as cells of the adaptive immune system. NLRs are critical regulators of major inflammation, cell death, immune and cancer-associated pathways. We used a data-driven approach to identify NLRs, AIM2 and NLR-associated gene expression and methylation patterns in low grade glioma and glioblastoma, using The Cancer Genome Atlas (TCGA) patient datasets. Since TCGA data is obtained from tumor tissue, comprising of multiple cell populations including glioma cells, endothelial cells and tumor-associated microglia/macrophages we have used multiple cell lines and human brain tissues to identify cell-specific effects. TCGA data mining showed significant differential NLR regulation and strong correlation with survival in different grades of glioma. We report differential expression and methylation of NLRs in glioma, followed by NLRP12 identification as a candidate prognostic marker for glioma progression. We found that *Nlrp12* deficient microglia show increased colony formation while *Nlrp12* deficient glioma cells show decreased cellular proliferation. Immunohistochemistry of human glioma tissue shows increased NLRP12 expression. Interestingly, microglia show reduced migration towards *Nlrp12* deficient glioma cells.

## Introduction

Gliomas account for 80% of primary malignant brain tumors. Based on the degree of malignancy, glioma are classified into low and high grade glioma^1^. Low grade glioma (LGG) specifically represents 40% of all central nervous system tumors in children. While majority of high grade glioma occur *de novo*, approximately 70% of the well differentiated, LGG progress into high grade, glioblastoma (GBM). GBM is multiforme in every aspect; grossly (increased necrosis), microscopically (pleomorphic nuclei, microvascular proliferation) and genetically (gene deletion, mutation), with a median survival of less than 15 months^2^. GBM forms 12–15% of all brain tumors and 50–60% of astrocytomas^3^. In spite of several multimodal treatment (radiation, surgery and chemotherapy) and advances including cancer immunotherapy, glioma prognosis remains poor^2^. Innate immune cells, including microglia and macrophages heavily infiltrate tumor microenvironment to regulate growth and progression^4^. Pattern recognition receptors (PRRs), including Toll-like receptors, C-type lectin receptors, RIG-I-like receptors, absent-in-melanoma (AIM)-like receptors (ALRs) and the nucleotide-binding and oligomerization domain, leucine-rich repeat containing receptors (NLRs), play a key role in tumor pathology as revealed by multiple human and murine studies^5,6^. NLRs are innate immune receptors sensing specific pathogen and damage-associated molecular patterns (PAMPs and DAMPs) and irritants, such as nucleic acids, flagellin and glucose, extracellular ATP, UV radiation^7^. NLR proteins regulate inflammation, cell death, proliferation, embryonic development, as well as transcriptional reprogramming of immune genes^8^. NLRs are expressed by cells of innate immunity, including monocytes, macrophages, dendritic cells, endothelial cells, and neutrophils, as well as cells of the adaptive immune system^9,10^. Once stimulated a subgroup of NLRs induce the assembly of a large multiprotein cytoplasmic complex called the *inflammasome* that includes a sensor protein (a NOD-like receptor, such as NLRP1, NLRP2, NLRP3, NLRP6, NLRP7, NLRC4, and NLRP12), an adaptor protein (ASC: apoptosis-associated speck-like protein containing a CARD domain), and caspase-1. In addition to NLR based inflammasomes, AIM2 (absent-in-melanoma 2) a member of the ALRs, is crucial for dsDNA induced inflammasome activation. Hoffman *et al*. first identified dysfunction of a NLR family member, *NLRP3* with a class of cryopyrin-associated periodic syndromes (CAPS)^11^. Dysregulated NLR function is associated with a wide array of diseases including microbial infections, diabetes, cardiac and metabolic disorders, autoimmune diseases and cancers^7^. *NLRP6* and *NLRP12* are negative regulators of canonical *NF-κB* and *MAPK*-dependent inflammatory signaling providing protection against colorectal cancer^12^. *In silico* studies, performed using The Cancer Genome Atlas (TCGA) and other pan-cancer data platforms confirm the pivotal role of NLRs in colorectal cancer^13^.

**Figure 1:**
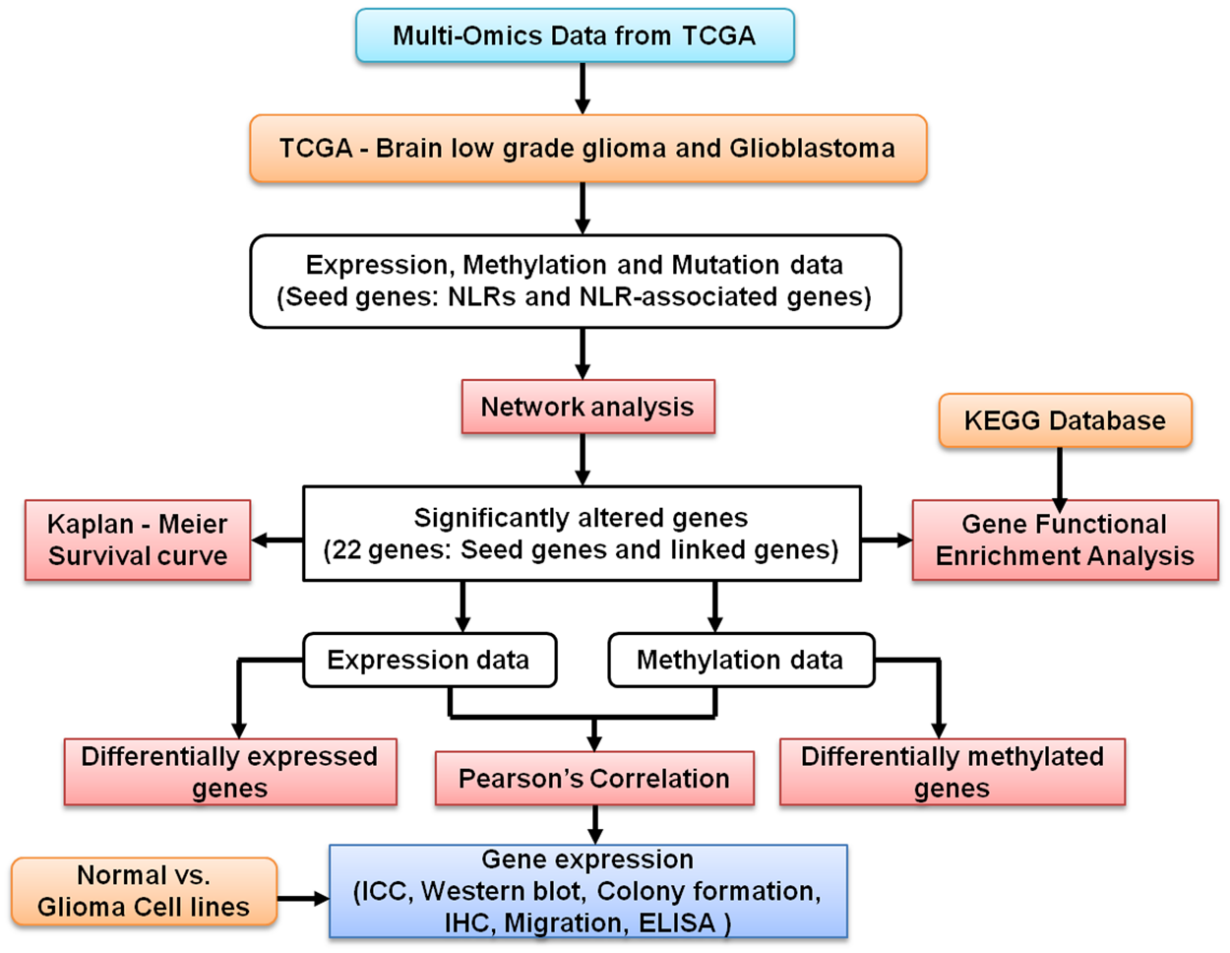
Schematic workflow of multi-dimensional investigation exploring the role of nucleotide-binding domain and Leucine rich-repeat containing receptors in glioma pathology.

Despite the critical role of NLRs in cancers, the physiological and functional significance of NLRs in gliomas remain largely unknown^14–17^. In this regard, our study provides basic insights into NLR and NLR-associated gene regulation in low grade glioma (LGG) and Glioblastoma (GBM), using TCGA datasets. A multi-omics approach utilizing both expression and methylation data, has been adopted in this study (Fig. 1). TCGA fulfills the importance of a systematic approach, high sample numbers, large comprehensive genomic profiles and clinical information. TCGA data is obtained from tumor tissue that comprises of multiple cell populations, such as glioma cells, endothelial cells and tumor-associated microglia/macrophages. To identify cell specific effects we carried out experimental studies utilizing cell culture and immunohistochemistry on human brain tissue. Our study utilizes bioinformatics and experimental data to understand the role of NLRs and NLR-associated genes in glioma pathogenesis (Supplementary Table 1). Importantly, our study is the first to report a differential regulation of NLRP12 in glioblastoma with differential cell specific roles. NLRP12 also known as Monarch-I and PYPAF7 is a pyrin-containing NLR protein. The gene was first identified and partially characterized in the HL60 human leukemic cell line^18^. NLRP12 has a tripartite domain structure with an N-terminal PYRIN domain, a central nucleotide binding site domain, and a C-terminal domain composed of atleast 12 leucine-rich repeat motifs^19^. The full-length human NLRP12 cDNA encodes for a 1062-aa protein with an estimated molecular weight of ~120 kDa. Alternative splicing results in multiple transcript variants of NLRP12^20^. Human NLRP12 is expressed predominantly in cells of myeloid lineage, such as neutrophils, eosinophils, monocytes, macrophages, and immature dendritic cells, and its expression is down-regulated in response to pathogens, pathogen products, and inflammatory cytokines^21,22^. However, the expression and functional analysis of NLRs including NLRP12 in glioma remains unknown.

## Materials and Methods

### Sample and data selection

The mRNA (RNA seq V2 RSEM) and gene expression (TCGA, provisional) data with z-score threshold of “±2.0”, was analyzed to obtain gene networks. The TCGA DNA methylation (Illumina Infinium Human Methylation450) and the RNAseq expression data (pancan normalized) for LGG and GBM, were downloaded using the UCSC browser. After filtering data, we have used samples with complete information for the genes of interest. We have used 226 - Grade 2, & 249 - Grade 3 and 172 – GBM samples for gene expression and methylation analysis.

### Generation of networks

The seed genes (NLRs and AIM2) were used to generate extended network using CBioPortal, that provides interactive analysis and visualization of networks altered in cancer^23,24^. The network consists of pathways and interactions from the Human Reference Protein Database (HPRD), Reactome, National Cancer Institute (NCI)–Nature, and the Memorial Sloan-Kettering Cancer Center (https://www.mskcc.org/) Cancer Cell Map (http://cancer.cellmap.org), as derived from the open source Pathway Commons Project. The genomic alteration is computed using multi-dimensional data of a particular cancer stored in TCGA. The portal automatically color codes edges by interaction type and overlays multi-dimensional genomic data onto each node, highlighting the frequency of alteration by mutation, copy number alteration (CNA), and mRNA up- or down-regulation. We have used LGG (262 samples) and GBM (135 samples) for the seed genes as input for the network analysis with respect to glioma. GBM and LGG networks were inclusive of genes with >10% alterations. Heat maps were constructed using complete linkage clustering method and heatmap.2, R function.

### Expression and methylation analysis

For gene expression and methylation data profiling, we used R version 3.3.2. Differentially expressed genes and methylated CpG loci were identified using Bioconductor limma and minfi. We used Kaplan-Meier method for estimating survival distribution of gene expression in glioma patients, using survival and prodlim, R package. We performed hazard ratio and multivariate analysis using the Cox regression model. The student’s t-tests were performed for determining significant group differences. We have drawn box plots for the significantly altered genes across the glioma grades – G2, G3 and G4, using ggplot2, R package and calculated corrected P-value using ANOVA statistics. The corrected P-value/posthoc tests were performed using Bonferroni method. Pearson’s correlation coefficient calculated significant correlations between the gene expression and methylation of glioma patient samples.

### Immunocytochemistry

We have used LN-18 (ATCC) human glioblastoma-derived cell line for the study. BV2 murine microglia, were a kind gift from Dr. Anirban Basu, NBRC, India. We purchased all reagents from Himedia and Sigma, unless specified otherwise. To simulate inflammation, cells were primed with lipopolysaccharide overnight (0.5 μg/mL; LPS) and immunolabeled by primary antibody (AIM2; NLRP12; ASC, Cell signaling; Caspase-1, Santa Cruz) incubation for overnight at 4 °C. The cells were washed and incubated with secondary antibody (Alexa-fluor 594/488- Life technologies) for 1 hour at room temperature (dark). DAPI (4′, 6-diamidino-2-phenylindole) stained nuclei blue.

### Immunohistochemistry

We used 5-μm paraffin-embedded sections that were deparaffinized and rehydrated through alcohols as described previously^25^. The paraffin embedded paraformaldehyde fixed glioma (grade 4, Glioblastoma) and normal brain tissue were obtained with approval from the Internal Review Board and the Ethics Committees of the All India Institute of Medical Sciences, Jodhpur and Tata Memorial Cancer Hospitals. We have acquired informed consent from human participants, regarding the use of tissue samples for experiments. We have performed all experiments in accordance with the ethical guidelines and regulations of the Indian Institute of Technology Jodhpur, All India Institute of Medical Sciences Jodhpur. For the detection of microglia, tissues were stained with Ricinus communis agglutinin-1 (RCA-1) lectin (Vector labs, FL-1081)^25^. Glioma (7 paraformaldehyde fixed paraffin embedded grade IV glioblastoma) and normal brain (2 paraformaldehyde fixed paraffin embedded) tissue sections were stained for NLRP12 using anti-NLRP12 antibody (GeneTex). Nuclei were stained blue with DAPI. The overlay shows co-localization of NLRP12 with microglia cell stain. Immunofluorescence was observed and analyzed using fluorescence microscope (Leica Systems) and ImageJ respectively^26^.

### Colony formation assay

Cells were seeded at a density of 40 cells per well (5% CO_2_; 37 °C) and small colonies were observed after 36–48 hours. For experiment, we added scrambled (Dharmacon) and NLRP12 (GeneTex) siRNA at concentrations −50 and100 nM, as per company protocol. Colonies were stained with Giemsa for 20 minutes, washed gently with distilled water and air-dried. Slides were observed using bright field microscope and images were captured using a cell phone camera. Results were quantified by counting number of colonies formed per well and cells present per colony.

### Western blot analysis

Microglial cells (BV2) and human glioblastoma cells (LN18) were seeded at a density of 5 × 10^5^ cells per well in a six-well plate. After 48hrs of transfection, cells were harvested and cell lysates were prepared in radio immunoprecipitation assay (RIPA) buffer containing freshly added protease inhibitor. Protein concentration was determined using Bradford’s Assay and 10ug of protein was loaded on to 12% SDS-polyacrylamide gel and transferred to nitrocellulose membrane. The membrane was blocked in 5% skimmed milk in TBST (Tris-buffered saline containing 0.05% Tween) and incubated with primary antibody for β-Actin (1:8000) and NLRP12 (1:5000). The membrane was washed with TBST and incubated with secondary IgG HRP conjugated antibody (1:15000). Protein expression was visualized using Azure Biosystems Gel Documentation system.

### Migration assay

Migration was assessed using Corning BioCoat™ Matrigel Invasion Chamber. 50,000 cells were seeded in serum free media in the inserts and incubated for 24hrs with the conditioned mediums. L929 conditioned media was taken as positive control and serum free media was taken as negative control. The effect of wild type LN18 and *siNlrp12* LN18 conditioned media on invasion capability of BV2 cells and vice-versa was analysed. The fixed cells were stained with Giemsa for observation under a bright-field microscope at 20X. Images were taken using mobile camera (Supplementary Fig. 7). The examiners were blindfolded for the experiment. Cells were manually counted for each image (membrane section). Total 8 sections were taken into consideration for each sample and average number of invaded cells per section was calculated.

### ELISA

To assess IL-6, IL-1β and TNF-α fresh cell free culture supernatants were analysed using Human IL-1β, IL-6 and TNF-α ELISA kits (BD Biosciences) (Supplementary Figs 8 and 9).

## Results

### Network analysis of NLR expression in LGG and GBM

**Figure 2:**
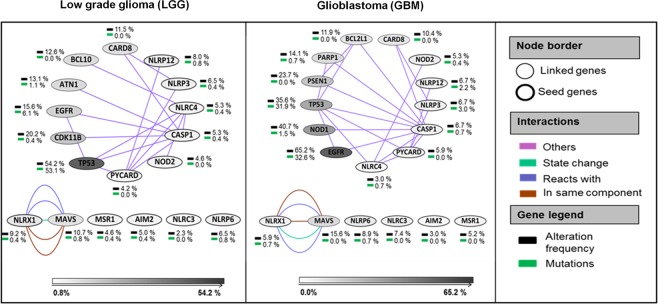
NLR gene expression network in low grade glioma and glioblastoma. Networks for TCGA Low grade glioma (LGG) and Glioblastoma (GBM), were generated using the cBioPortal platform. The edges are color coded by interaction type. Gene legend highlights the frequency of alteration (inclusive of copy number alterations, mutations and mRNA regulation) (black) and mutations (green) with respect to the LGG and GBM associated pathways.

The cellular and molecular complexity of glioma and cross talk within the tumor microenvironment bring focus on genomic and epigenetic variations occurring in glioma. To understand the importance of NLRs and their interactions in glioma, we used a multi-dimensional approach (Fig. 1). We started our analyses by extracting LGG and GBM patient data sets from The Cancer Genome Atlas (TCGA)^27^. The glioma networks were generated using the cBioPortal platform for visualization, analysis and download of large-scale cancer genomics data sets^23,24^. Networks were simplified using Cytoscape, an open source software for integrating biomolecular interaction networks with high-throughput expression data into a unified conceptual framework^28^. The ***seed genes*** (genes of interest) included *NLRP3*, *NLRP6*, *NLRP12*, *NLRC3*, *NLRC4*, *NLRX1*, *PYCARD*, *CASP-1*, *AIM2*, *MSR1* and *NOD2* (Fig. 2). Glioma networks provided information about genes that were highly altered as per the underlying data information. Some other genes including *TP53*, *EGFR*, *NOD1*, *CDK11B*, *MAVS*, *BCL10*, *BCL2L1*, *PARP1*, *PSEN1*, *CARD8*, and *ATN1* also emerged through network analysis (***linked genes***). These genes have important functional roles in major DNA damage repair, cell proliferation, cell death, tumor-suppressor and other core cell signaling pathways^29–31^. In the present study, we have generated an extended network using seed genes (*NLRs* and *AIM2*) in glioma. The network provides an overview of the altered genes in pathways related to glioma highlighting the frequency of alteration by mutation, CNA, and mRNA up- or down-regulation. Further, DNA methylation and gene expression data were used to understand the degree of alteration of the seed genes in glioma. The network was altered in 38.9% cases for LGG and in 38.5% cases for GBM (Fig. 2). Most frequently altered genes in LGG were *TP53* (54% altered); *EGFR* and *CDK11B* (15 to 20% altered); AIM2, *NLRP6*, *CASP1*, *NLRP3*, *NLRC4*, *NLRP12*, *CARD8*, *BCL10*, *ATN1*, *NLRX1* and *MAVS* (5 to 10% altered). Notably, *TP53* (53% mutated) and *EGFR* (6% mutated) were the most frequently mutated driver genes across LGG. The GBM network shows *EGFR* (65% altered), *NOD1* (40% altered) and *TP53* (35% altered) as most frequently altered genes. As expected, *EGFR* (32.6% mutated) and *TP53* (31.9% mutated) came up as highly mutated driver genes for GBM. Interestingly, *TP53* was more frequently altered and mutated in LGG as compared to the GBM and, *EGFR* was more frequently altered and mutated in GBM as compared to the LGG. *EGFR* and *TP53* alterations are known to contribute significantly in various tumors, including glioma^32,33^. Other frequently altered genes were *MAVS* (15% altered) and *PSEN1* (23% altered); *CARD8*, *PARP1* and *BCL2L1* (10–15% altered); *MAVS*, *NLRC3*, *NLRX1*, *NLRP6*, *PYCARD*, *CASP1*, *NLRP3*, *NLRP12* and *NOD2* (5–10% altered).

### Differential expression of NLRs in LGG and GBM

The World Health Organization (WHO) has classified glioma into four grades, depending on the degree of malignancy^1^. Based on histology, LGG stratifies into grade 2 and grade 3 glioma. The grade 2 and 3 glioma, are further divided into three histological types - astrocytoma, oligoastrocytoma, and oligodendroglioma. Grade 4 glioma, is a highly aggressive and advanced form of glioma, known as glioblastoma (GBM). Unfortunately, there is no clear distinction between different grades of glioma, based on their histology. Cancer results from genomic alterations, including copy number variation, mutation and methylation^34^ and so forth. Gene list obtained through network analysis, (11 ***seed*** and 11 ***linked***) underwent quantitative genomic analysis (Fig. 1). The expression of genes list, including NLRs in LGG and GBM, was visualized using heat map representation (Supplementary Fig. 1). We could see overlapping gene expression profiling of grade 2, and 3 LGG samples (Supplementary Fig. 1(a)). Interestingly, we observed characteristic gene expression clusters for GBM with respect to both grade 2 and 3 of LGG (Supplementary Fig. 1(b,c)).

**Figure 3:**
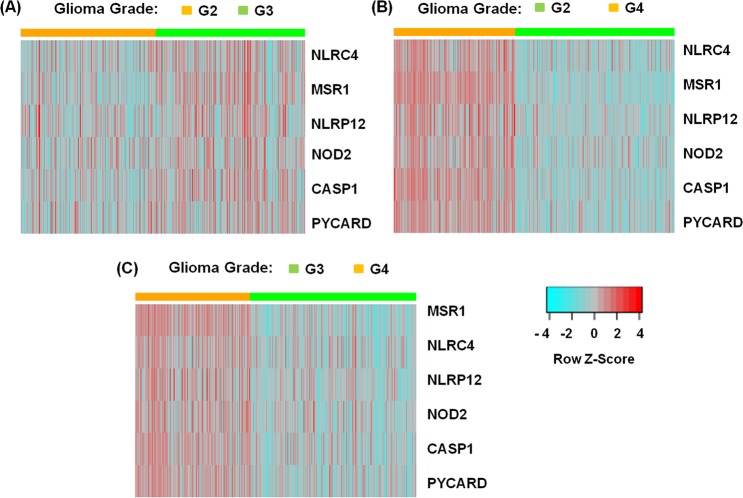
Heat map clustering representation for most significantly differentially expressed NLR-associated genes in glioma. We have grouped all the glioma samples by glioma grade 2, 3 and 4. Here, G2, G3 and G4 represents grade 2, grade 3, and grade 4 respectively. (**A**) Shows gene expression of most differentially expressed genes, *MSR1*, *NLRC4*, *NLRP12*, *NOD2*, *CASP1* and *PYCARD* across the grade 2 (orange) & grade 3 (green) of LGG samples. Similarly, panel (**B**,**C**) shows characteristic gene expression clusters between grade 4 (orange) & grade 2 (green) and grade 4 (orange) & grade 3 (green) glioma samples respectively. The segregation of grade 4 glioma from the grade 2 and grade 3 glioma is evident as the resulting heatmaps form expression ‘blocks’. Here, relative up-regulated and down-regulated gene expression, are shown in red and blue respectively.

**Figure 4:**
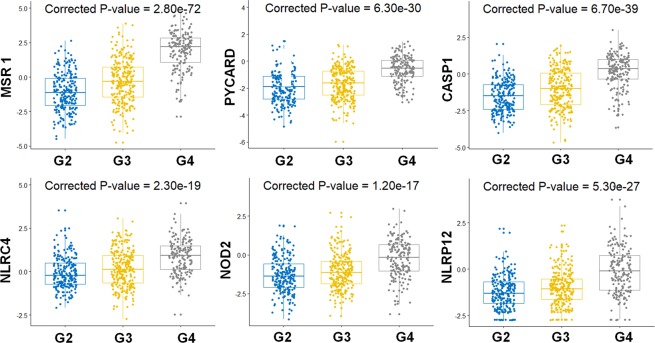
Differential *NLR gene expression in different grades of Glioma*. The box plots show average gene expression values for differentially expressed genes, *MSR1*, *CASP1*, *PYCARD*, *NLRC4*, *NOD2* and *NLRP12* in LGG – grade 2 & grade and GBM- grade 4 glioma. Here, G2, G3 and G4 represents grade 2, grade 3, and grade 4 respectively. We conducted one-way ANOVA followed by posthoc tests using Bonferroni method, to calculate the corrected P-values for the differentially expressed genes in different grades of glioma.

To further understand and quantify the NLR gene expression pattern in glioma, we performed differential gene expression analysis across LGG and GBM. As observed earlier from heat maps, we did not see significant differential gene expression across the grade 2 and 3 LGG (Table 1). The distribution of gene expression in grade 4 is significantly different from that of grade 2 & grade 3. However, we found *MSR1*, *NOD2*, *NLRP12*, *NLRC4*, *PYCARD and CASP1* as the most significantly differentially expressed genes (log_2_ fold change - greater than or equal to 1) in GBM with respect to LGG. The differential gene expression grouped by grade is being visualized using heat map representation (Fig. 3). The box plots show significant differential expression of genes in G2, G3 and G4 grades of glioma. Here, we have conducted ANOVA and posthoc test analysis using Bonferroni method to calculate the corrected P-value for each gene (Fig. 4). Due to very less or no heterogeneity of grade 2 and grade 3, the samples are not very well segregated and clear overlapping clustering of samples is observed. Whereas, samples are better segregated in grade 2 vs. grade 4 and in grade 3 vs. grade 4 glioma patient samples (Figs 3 and 4).

**Table 1:** Differential expression of NLRs and NLR-associated genes in glioma.

Between groups	Grade 3 vs. Grade 2		Grade 4 vs. Grade 2		Grade 4 vs. Grade 3	
Gene	log_2_FC	Adjusted	log_2_FC	Adjusted	log_2_FC	Adjusted
		p-value		p-value		p-value
**MSR1**	**0**.**79**	4.97E-07	**3**.**00**	8.41E-69	**2**.**22**	2.09E-39
TP53	0.37	9.35E-06	0.76	1.07E-17	0.39	1.54E-06
BCL10	0.19	6.29E-05	0.82	1.00E-57	0.63	3.33E-35
**CASP1**	**0**.**48**	1.48E-04	**1**.**70**	2.03E-40	**1**.**23**	7.32E-20
NOD1	0.26	3.02E-04	0.70	1.34E-18	0.43	9.23E-08
**NLRC4**	**0**.**27**	1.01E-02	**0**.**95**	1.28E-20	**0**.**68**	2.77E-10
CARD8	0.16	1.01E-02	0.30	3.50E-07	0.14	1.73E-02
EGFR	0.45	1.01E-02	0.87	1.22E-05	0.41	7.10E-02
PSEN1	−0.11	1.01E-02	−0.13	2.38E-03	−0.02	6.90E-01
MAVS	0.09	1.01E-02	−0.16	4.30E-04	−0.26	6.16E-10
**NLRP12**	**0**.**22**	1.76E-02	**1**.**16**	8.98E-23	**0**.**94**	9.18E-16
**PYCARD**	**0**.**26**	2.68E-02	**1**.**32**	3.18E-29	**1**.**06**	1.21E-19
**NOD2**	**0**.**21**	6.57E-02	**1**.**03**	3.27E-16	**0**.**82**	2.62E-11
ATN1	−0.07	1.20E-01	−0.67	3.92E-34	−0.60	2.79E-28
NLRP6	0.17	1.20E-01	0.16	1.67E-01	−0.01	8.98E-01
CDK11B	0.05	2.53E-01	−0.16	4.30E-04	−0.21	2.11E-06
BCL2L1	0.04	4.36E-01	0.18	8.77E-05	0.14	3.57E-03
PARP1	0.02	5.46E-01	−0.23	2.61E-08	−0.26	1.10E-11
NLRC3	−0.03	5.69E-01	−0.32	7.34E-09	−0.29	3.38E-07
AIM2	−0.05	6.89E-01	−0.13	3.31E-01	−0.08	6.15E-01
NLRX1	−0.02	7.48E-01	−0.38	1.14E-12	−0.37	5.35E-11
NLRP3	−0.03	8.08E-01	−0.10	3.50E-01	−0.07	6.15E-01

Abbreviations: FC, fold change.

MSR1 emerged as most significantly overexpressed gene in GBM, with high fold change (positive) value with respect to LGG. Here, increased differential gene expression of *MSR1*, *BCL10*, *NOD1*, *NOD2*, *NLRP12*, *NLRC4*, *TP53* and *EGFR* gene expression in GBM indicates altered innate immune signaling and other core cell signaling pathways in glioma pathogenesis. Importantly, tumor-associated macrophages signature comprising of distinct M2-macrophage related gene - *MSR1* (macrophage-specific integral membrane glycoprotein), are highly enriched in glioblastoma tumors^35^. In GBM, *EGFR* overactivation triggers activation of multiple downstream signaling pathways such as PI3K/Akt/rapamycin-sensitive mTOR pathway, followed by poor prognosis and drug resistance^36^. Similarly, *NOD1* activation promotes colon cancer growth and metastasis^37^. *TP53*, tumor suppressor gene creates a complex signaling network via significant associations with cell cycle, DNA repair, apoptosis, angiogenesis and metabolic pathways^33,38^.

### NLR gene methylation in LGG and GBM

**Figure 5:**
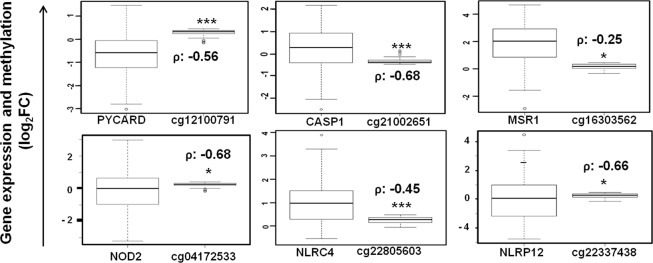
Negative regulation of NLR gene expression by methylation in Glioma. The box plots show significant inverse correlation between expression of *NLRs* and *NLR*-associated genes and differential methylation levels. Here, ρ denotes correlation coefficient calculated by the Pearson’s correlation formula.

Targeting DNA methylation of specific biomarker gene promoter regions such as *MGMT* methylation has undoubtedly favored glioma prognosis and improved survival^39^. In this regard, we studied the genome-wide importance of methylation by analyzing CpG loci methylation in seed genes and linked genes, using same patient samples of TCGA- GBM and LGG. Table 2 shows differentially methylated CpG loci in grade 4 vs. grade 2 and grade 4 vs. grade 3 glioma respectively. Notably, we observed highly significant negative correlation between gene expression and methylation levels using the Pearson’s correlation coefficient, in case of GBM (Table 2). Highly significant inverse correlation for the differentially expressed genes is as depicted through box-plots (Fig. 5). Stone *et al*., first identified aberrant promoter methylation-induced suppression of *PYCARD* expression in human glioblastoma^40^. *PYCARD*, also known as apoptosis-associated speck-like protein containing CARD (*ASC*) is involved in several cell death-associated pathways and methylation-induced *PYCARD* silencing occurs across multiple cancers^17,40^. *ASC* also mediates NLR inflammasome formation upon activation through various PAMPs and DAMPs^7^. Our findings confirm high inverse correlation between *ASC* expression and methylation levels in case of GBM. Interestingly, we found differential expression and significantly methylated CpG loci for *NLRP3* (cg21991396, cg07313373) and *CASP1* (cg21002651, cg13802966) in GBM. Recently, Paugh *et al*., observed significantly high *CASP1* and *NLRP3* expression in glucocorticoid resistant leukemia cells, due to significantly lower somatic methylation of same *CASP1*(cg13802966) and *NLRP3* (cg21991396) promoter regions^41^. We found significant inverse correlation between methylated CpG loci and expression in GBM, for genes - *AIM2*, *CASP1*, *EGFR*, *MSR1*, *NLRC3*, *NLRC4*, *NLRP3*, *NLRP12*, *NLRX1*, *NOD1*, *PYCARD*, and *CDK11B* (Table 2).

**Table 2:** Differentially methylated CpG loci for NLRs and NLR-associated genes in glioma.

Gene	CpG loci	Grade 4 vs. Grade 2		Grade 4 vs. Grade 3		Distance from TSS	Grade 4	
		log_2_FC	adjusted	log_2_FC	adjusted		correlation coefficient (ρ)	p-value
			p-value		p-value			
AIM2	cg11003133	−0.23	3.96E-56	−0.25	6.88E-64	256	−0.26	4.42E-02
	cg00490406	−0.09	2.29E-25	−0.09	1.92E-27	−126	−0.34	6.52E-03
ATN1	cg09215316	0	1.36E-02	−^A^	—	3	−0.24	5.90E-02
	cg11831988	—	—	0	1.48E-01	53	−0.36	3.74E-03
BCL2L1	cg08619561	0	7.40E-07	—	—	47	−0.24	6.12E-02
	cg02457826	—	—	0	4.10E-02	−77	−0.27	3.05E-02
BCL10	cg06913958	−0.33	3.15E-53	−0.33	3.82E-57	−2054	−0.43	4.76E-04
**CASP1**	**cg21002651**	**−0**.**32**	**5**.**56E-47**	**−0**.**35**	**3**.**20E-56**	**−49**	**−0**.**68**	**1**.**49E-09**
	**cg13802966**	**−0**.**2**	**1**.**28E-42**	**−0**.**24**	**6**.**10E-58**	**6**	**−0**.**39**	**1**.**94E-03**
EGFR	cg18809076	−0.25	2.35E-23	−0.32	8.60E-37		−0.76	1.21E-12
	cg14344486	−0.06	1.91E-18	−0.07	2.96E-22	−25141	−0.81	1.38E-15
**MSR1**	**cg16303562**	**−0**.**19**	**2**.**36E-36**	**−0**.**2**	**3**.**81E-39**	**−33**	**−0**.**25**	**5**.**12E-02**
NLRC3	cg00011564	−0.04	1.70E-12	−0.04	1.76E-14	−7473	−0.28	2.66E-02
	cg04082551	−0.04	6.82E-08	−0.04	1.39E-08	−7605	−0.28	3.05E-02
**NLRC4**	**cg22805603**	**−0**.**12**	**1**.**35E-29**	**−0**.**12**	**4**.**41E-34**	**46**	**−0**.**45**	**2**.**67E-04**
	cg07055315	−0.19	2.27E-17	−0.22	2.38E-24	−23	−0.26	4.45E-02
NLRP3	cg07313373	−0.23	2.55E-43	−0.25	2.52E-49	−326	−0.36	4.15E-03
	cg21991396	−0.11	1.47E-23	−0.12	2.14E-25	63	−0.35	5.64E-03
**NLRP12**	**cg07042144**	**−0**.**1**	**9**.**73E-23**	**−0**.**11**	**4**.**23E-26**	**244**	**−0**.**6**	**2**.**62E-07**
	**cg22337438**	**−0**.**08**	**6**.**47E-13**	**−0**.**08**	**3**.**46E-14**	**211**	**−0**.**66**	**4**.**55E-09**
NLRX1	cg26863308	−0.14	3.99E-10	−0.18	6.10E-16	611	−0.44	3.17E-04
	cg24516766	—	—	0	1.68E-01	—	−0.57	1.56E-06
NOD1	cg04071779	−0.35	4.10E-48	−0.38	4.23E-59	766	−0.35	5.61E-03
	cg09579281	−0.07	9.46E-17	−0.08	1.16E-23	3107	−0.32	1.05E-02
**NOD2**	**cg16771652**	**−0**.**1**	**1**.**81E-22**	**−0**.**1**	**1**.**02E-26**	**−664**	**−0**.**6**	**2**.**70E-07**
	**cg04172533**	**−0**.**06**	**7**.**52E-12**	**−0**.**07**	**1**.**11E-13**	**−1441**	**−0**.**68**	**1**.**39E-09**
**PYCARD**	**cg05907835**	**−0**.**11**	**5**.**31E-17**	**−0**.**11**	**1**.**10E-17**	**−249**	**−0**.**54**	**6**.**28E-06**
	**cg12100791**	**−0**.**06**	**1**.**30E-10**	**−0**.**06**	**3**.**08E-12**	**−320**	**−0**.**56**	**1**.**93E-06**
CDK11B	cg21921584	−0.03	1.00E-05	−0.04	4.81E-08	3891	−0.35	5.23E-03
	cg09283376	0.01	3.04E-03	0.02	5.56E-05	—	−0.43	4.83E-04
PSEN1	cg13173405	−0.01	9.07E-02			−191	−0.39	1.61E-03
	cg26376566	—	—	0	5.11E-02	—	−0.38	2.36E-03

Abbreviations: FC, fold change; TSS, transcription start site. ^A^Denotes log_2_FC insignificant.

### Prognostic value of NLRs and NLR-associated genes in glioma

**Figure 6:**
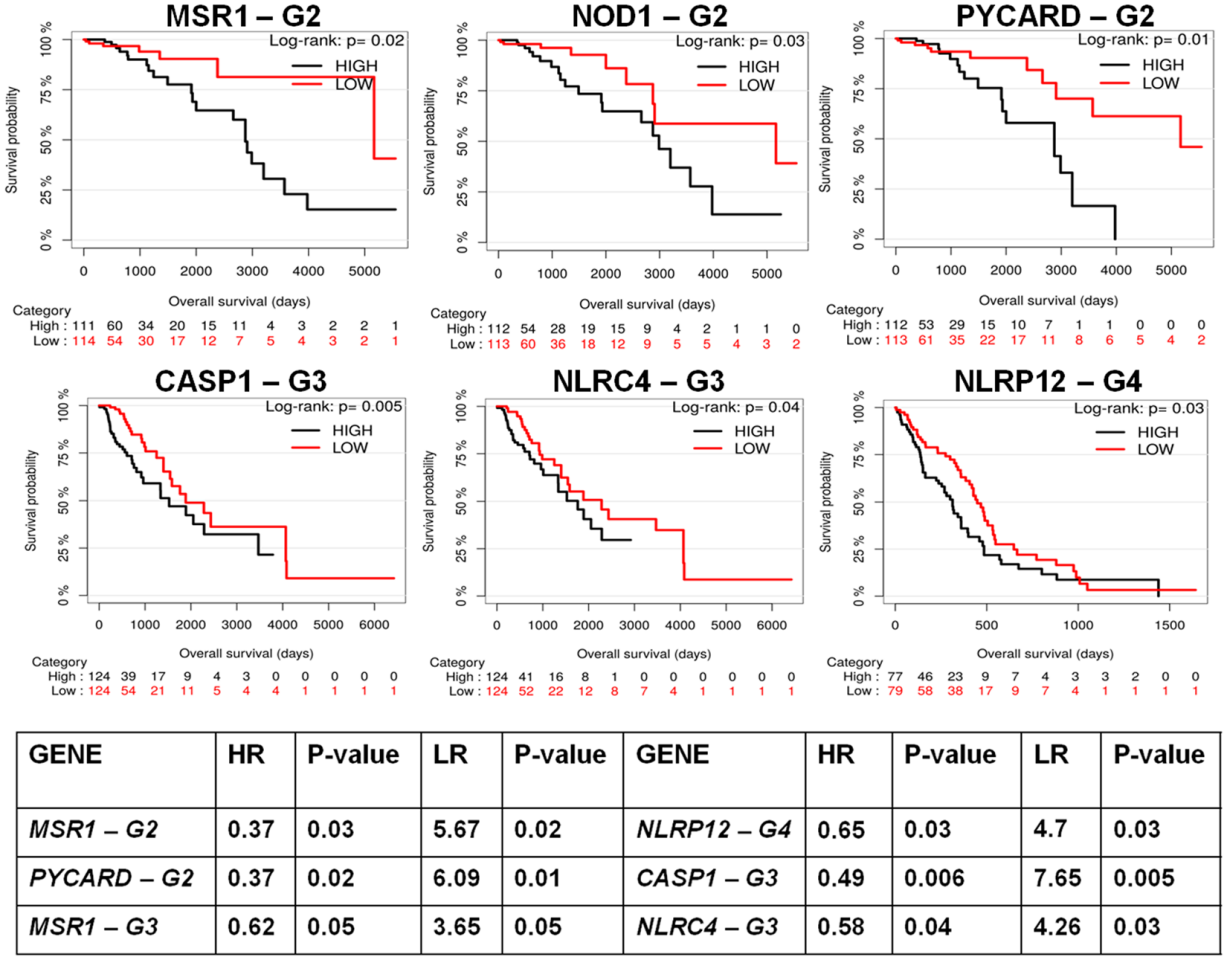
Kaplan-Meier survival curves of low grade glioma and glioblastoma patients stratified by the expression of NLRs. The survival curves show significant association of NLRs and other related genes with patient survival. Here, G2, G3 and G4 represents grade 2, grade 3, and grade 4 respectively. The table (below) represents hazard ratio (HR) and Likelihood ratio (LR) for most significantly expressed genes in glioma. Here, the P-values indicate level of significance of the HR and LR calculated for the corresponding gene.

Gene expression profiling helped in identifying association of differentially expressed genes with early prognosis and clinical outcome in GBM patients. We used Kaplan-Meier method to assess the prognostic value of the corresponding genes in low grade glioma (LGG) and glioblastoma (GBM). To evaluate the biomarker in several conditions, we selected relevant glioma patient TCGA samples and corresponding clinical information. Patient samples of Grade 2 and 3 LGG were placed into first and second category, and grade 4 GBM into the third category. We calculated patient overall survival (OS) based on gene expression and stratified patients based on death information (Fig. 6). For each gene, expression set was divided into two categories based on the median expression value. For all grades, patients (grade 2, n = 226; grade 3, n = 249; grade 4, n = 172) were grouped into high-expression group (black curve) and low-expression group (red curve) for a gene of interest. From Kaplan-Meier survival analyses, we observed that the differentially expressed genes are highly correlated with glioma patient survival. In fact, *NLRP12* (P < 0.03), *PYCARD* (P < 0.01), *CASP1* (P < 0.005), *MSR1* (P < 0.02), *NOD1* (P < 0.03) and *NLRC4* (P < 0.04) genes significantly separate the two risk groups characterized by differences in their gene expression. It is seen that high expression of these genes leads to poor overall survival of the patients. The above identified genes, *NLRP12*, *PYCARD*, *CASP1*, *MSR1*, *NOD1* and *NLRC4* perform specific tumor regulatory roles in cancers^7,42,43^. MSR1 has been reported to play important regulatory roles in glioma pathology utilizing single cell profiling of human gliomas^44^. However, the roles of *CASP1*, *NOD1*, *NLRC4* and *NLRP12* are not reported in relation to glioma. Since, majority of grade 4 glioma occur de novo and approximately 70% of the grade 2 and 3 glioma progress into glioblastoma. There is significant need for drugs targeted towards specific glioma grade. The differentially expressed genes show significant association with overall survival of specific glioma grade patients, emerging as promising biomarkers for prognostically significant molecular sub-typing of LGG and GBM. Interestingly, for GBM, *NLRP12* significantly separates the two risk groups characterized by differences in their gene expression. We also identified that *ATN1*, *CARD8*, *BCL10*, *EGFR* and other genes contribute significantly to low overall survival rate of the grade 3 patients (Supplementary Fig. 2).

To support our findings, we assessed our genes list for survival analyses using additional glioma database, REMBRANDT. Though the impact of *CARD8*, *CASP1*, *MSR1*, *PYCARD* and *PARP1*gene expression on overall survival (OS) was statistically significant for LGG, most of the prognostic values were different as compared to the TCGA dataset (Supplementary Fig. 3). The REMBRANDT dataset classifies the glioma samples based on the revised molecular subtyping and WHO classification of CNS tumors^1^. The clinical information shows that the REMBRANDT datasets has heterogeneous sample size and patient population as compared to the TCGA. Next, we have performed pan-cancer analysis for above identified genes using different TCGA cancer datasets- Colon adenocarcinoma, Lung adenocarcinoma, and Head and neck squamous cell carcinoma. We did not find any significant association of these genes with the other cancer types, using the Kaplan-Meier survival analyses (Supplementary Table 2). The results confirm strong association between the above-identified differentially expressed genes and survival outcome is specific to LGG and GBM.

We have further assessed the gene prognostic values using multivariate hazard ratio (HR) and likelihood ratio (LR) analyses using Cox regression model. We have performed Cox regression analysis based on gene expression profiles and stratified patients based on death information (Fig. 6- Table). We have observed high HR (greater or equal to 0.5) for *NLRC4*, *CASP1*, *NLRP12* and *MSR1* genes in grade 3 & grade 4 glioma. The LR results also coincide with the HR values obtained for these genes. We have found high LR (greater or equal to 5) for the differentially expressed genes. The P-values obtained for both HR and LR of these genes are low and statistically significant. Therefore, the differentially expressed genes identified in our study may have an increased modulatory effect in glioma pathology. Based on high significance level, we suggest *NLRP12* as possible prognostic marker for glioblastoma.

### NLRP12 regulates cellular proliferation *in vitro* in GBM

**Figure 7:**
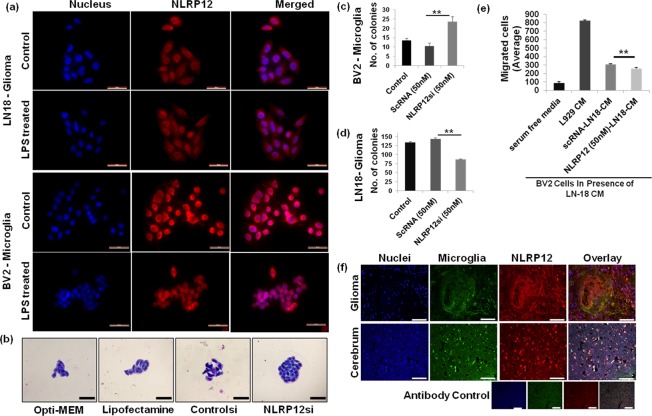
NLRP12 inhibition regulates cellular proliferation. (**a**) NLRP12 expression was observed in glioma (LN18) and microglial (BV2) cells using immunofluorescence (magnification: 40X, scale bar: 50 μm). (**b**) Colony formation assay shows increased cellular proliferation in the NLRP12si treated microglia. Magnification: 40X. (**c**,**d**) Quantification of both BV2 and LN18 cells per colony. (**e**) Effect of LN18 (NLRP12 siRNA treated) cell conditioned medium (CM) on BV2 cell migration was assessed using migration assay. 8 image sections per sample were quantified for migration analysis. Student’s t-test and one-way AVOVA were performed to find the statistical significance of colony formation and migration assay respectively (p-value; *<0.05, **<0.005). The error bar indicates standard error of mean. (**f**) NLRP12 expression in human normal brain tissue and glioma sections, using anti-NLRP12 antibody (N = 7). Nuclei were stained blue with DAPI and microglia were stained green with FITC tagged lectin RCA (Ricinus Communis Agglutinin-1); Magnification: 20X, scale bar: 100 µm.

TCGA provides a comprehensive genome profiling from the whole tissue, thereby neglecting individual cell population effects. However, growing evidence suggests cell and tissue-specific roles of NLR in cancers^5,12^. Therefore, we looked at the expression of differentially expressed genes, *ASC/PYCARD*, *AIM2*, and *CASP1* under normal and inflammatory conditions in microglia and glioma cells (Supplementary Figs 4–5). BV2, microglia and LN-18 glioma cells are known to secrete various proinflammatory cytokines upon LPS stimulation. The response varies significantly with LPS dose and exposure time^45–49^. *NLRP12* regulates non-inflammasome and anti-inflammatory signaling by both canonical and non-canonical NF-κB pathway inhibition^12^. Our TCGA glioma findings show significant differential *NLRP12* gene regulation and high prognostic value, which motivated us to examine the expression and functional association of *NLRP12* with glioma. Using immunofluorescence, we have characterized NLRP12 expression in LN18 glioma and BV2 microglia cell lines (Fig. 7a). To understand the role of NLRP12 in microglia and glioma cell proliferation, we performed colony formation assay utilizing *NLRP12* siRNA (Supplementary Fig. 6). Bright field imaging and quantitative analysis of Giemsa-stained microglia shows increased colony formation upon *NLRP12* inhibition (Fig. 7b,c) while LN18 glioma cell showed reduced proliferation (Fig. 7d). These finding suggest NLRP12 inhibition leads to increased microglial proliferation and reduced glioma cell proliferation *in vitro*. NLRP12 has been previously shown to control dendritic and myeloid cell migration to affect contact hypersensitivity^50^. We tested migration of microglia and glioma cells towards conditioned media from glioma cell or microglia cells derived from *Nlrp12* deficient cells. Wild type BV2 microglia showed significantly reduced migration towards *Nlrp12* deficient LN18 glioma cell culture supernatants in comparison to conditioned media from scrambled siRNA treated LN18 glioma cell culture supernatants (Fig. 7e). L929 cell supernatant (containing M-CSF) was used as a positive control for migration^51^. The reverse experiment with si*Nlrp12* treated BV2 conditioned media did not show any effect (Supplementary Fig. 7). Additionally, staining of human glioma and normal brain tissue showed colocalization of NLRP12 with microglia (Fig. 7f). These preliminary findings suggests cell specific role of NLRP12 in glioma as indicated by the colony forming assay, immunocytochemistry and immunohistochemistry experiments.

## Discussion

Cancer cells devise elaborate mechanisms to evade immune surveillance and consequent elimination. Immune system subversion is an area of intense focus for classifications of cancers and development of directed cancer therapeutics. The NLR family of receptors have been recognized as important regulators of immunity and inflammation^5,52,53^. NLRs are expressed by cells of innate immunity, including monocytes, macrophages, dendritic cells, endothelial cells, and neutrophils, as well as cells of the adaptive immune system^9,10^. NLRs have been extensively studied in inflammation-associated colon carcinogenesis. *Nlrp3*^−/−^, *Nlrp6*^−/−^, *Nlrc4*^−/−^, *Nlrp1*^−/−^, *Nlrx1*^−/−^ and *Nlrp12*^−/−^ mice show increased susceptibility to inflammation-induced colorectal cancer as compared to wild-type mice^17^. Given the pivotal role of NLRs in immunity and inflammation, understanding the role of NLRs in cancer allows for development of therapeutic strategies and rational drug design.

Gliomas form a class of recalcitrant, untreatable tumors with high morbidity and mortality. Surgery and radiotherapy in combination with classical alkylating agents such as temozolomide offer little hope from the poor prognosis^54^. There have been some investigations into the prognostic markers for gliomas including Poly (ADP-ribose) polymerase-1 (*PARP-1*) Val762Ala polymorphism. The subgroup analysis of cancer types revealed that the –762Ala allele was associated with increased risk of gastric, cervical, and lung cancers and a decreased risk of glioma^55^. Wang *et al*., utilized the overexpression of IL-13Rα2 on glioblastoma to design tumor specific therapeutics. The authors used Pep-1-conjugated PEGylated nanoparticles loaded with paclitaxel (PTX) as an effective drug delivery system through IL-13Rα2 mediated endocytosis in treatment of GBM^56^. Gliomas are infiltrated with immune cells and the contribution of NLR signaling in glioma pathogenesis remains largely unknown. In this study, we have used a multipronged, computational and experimental approach to, (1) mine LGG and GBM- TCGA data for NLR expression, and (2) methylation-dependent gene regulation in glioma, (3) correlate NLR expression with patient survival, (4) analyze differential expression of NLRs in glioma cell lines, and (5) functionally characterize NLR dependent effects on colony formation, an important characteristic of tumors. Our study identifies NLRP12 as a candidate prognostic marker for glioma progression.

Human NLRP12 is expressed predominantly in cells of myeloid lineage, such as neutrophils, eosinophils, monocytes, macrophages, and immature dendritic cells, and its expression is down-regulated in response to pathogens, pathogen products, and inflammatory cytokines^21,22^. NLRs perform both pro-tumorigenic and anti-tumorigenic cell and tissue-specific roles in cancer^43^. However, the expression and functional significance of NLRs including NLRP12 in glioma remains unknown. Rare mutations in *NLRP12*, are associated with periodic fevers in humans^57^. Nonsense and splice mutations within human *NLRP12* diminish suppression of NF-kB signaling; however, some variants do not exhibit such activity and are associated with modestly enhanced or more rapid inflammasome activation. NLRP12 has been implicated as a negative regulator of the canonical and non- canonical pathways of NF-kB signaling^58^. NLRP12 in hematopoietic progenitor cells, serves as a critical checkpoint for osteoclast development to limit tumor necrosis factor (TNF)-induced apoptosis^59^. TNF reduces brain tumor growth by enhancing macrophage recruitment and microcyst formation^60^. This is in line with our results where NLRP12 inhibition in microglia leads to increased colony formation indicative of a tumor like phenotype. Moreover, *Nlrp12* deficient glioma cells show lack of growth. The literature for NLRP12 regulation of IL-1beta secretion has been somewhat conflicting. While activated dendritic cells from Nlrp12‐deficient mice displayed normal IL‐1β secretion^50^, activated THP‐1 cells transduced with NLRP12 small interfering RNA were shown to secrete increased levels of IL‐1β^61^ and NLRP12 expressed in COS‐7L cells was found to activate proIL‐1β secretion^62^. Also, NLRP12 in colon associated cancer and colitis serves as a negative regulator of the canonical and non- canonical pathways of NF-kB signaling^58^.

NLRP12 is known to exhibit cell and tissue-specific roles in cancer^58,63,64^. NLRP12 protein expression is decreased in colorectal cancer (CRC) tissues compared to the surrounding normal tissue of the CRC patients. NLRP12 down-regulation leads to increased migration, proliferation and drug-resistance capacity of CRC (HCT116) cells *in vitro* (Pan *et al*., 2018). Enhanced immune cell infiltration and pro-inflammatory cytokine production leads to prolonged colon inflammation and increased tumorigenesis in the Nlrp12-deficient mice (Zaki *et al*., 2011). NLRP12 attenuates inflammation through negative regulation of NF-κB and ERK activation in macrophages. NLRP12 signaling in the hematopoietic cells is critical for protection against colon carcinogenesis. NLRP12 also attenuates colon inflammation by regulating gut microbiota^65^. Nlrp12 deficiency decreases microbiome diversity and increased susceptibility to colitis. Study by Karan *et al*., shows implication of NLRP12 inflammasome in prostate cancer^66^. Contrary to colon cancer, NLRP12 expression is significantly higher in malignant prostate as compared to their adjacent benign tissues. Increased NLRP12 expression associates with the progression of prostate cancer suggesting NLRP12 as potential marker to treat colorectal cancers. These preliminary findings show distinct cell- specific expression and regulation of NLRP12 in multiple cancers. SSFA2, also known as KRAP (Ki-ras-induced actin-interacting protein) shows higher expression in GBM than normal brain tissues. Global gene expression profiling of glioma (U87MG) cells shows, SSFA2 serves as strong activator of the Nlrp12 inflammasome^67^. In line with our results, Zhu *et al*., also found SSFA2 deletion inhibits glioma cell proliferation and increased cancer cell apoptosis. Our study merits further investigations *in vivo* models of glioma initiation and progression to further tease the relative contribution of NLRP12 at a cellular and molecular level in the heterogeneous population of glioma.

## Supplementary information

Differential Expression Profile of NLRs and AIM2 in Glioma and Implications for NLRP12 in Glioblastoma

## Data Availability

The TCGA glioma datasets analysed are freely accessible and available for download through the cBioPortal platform for cancer genomics (http://www.cbioportal.org/). The datasets analysed during the current study are also available from the corresponding author on reasonable request.
