# Supplementary material for: Differential Expression Profile of NLRs and AIM2 in Glioma and Implications for NLRP12 in Glioblastoma
Source: Sci Rep. 2019 Jun 11;9:8480. doi: 10.1038/s41598-019-44854-4 (PMC6559951; doi:10.1038/s41598-019-44854-4)
Supplement: Supplementary file 1 — Differential Expression Profile of NLRs and AIM2 in Glioma and Implications for NLRP12 in Glioblastoma [file 41598_2019_44854_MOESM1_ESM.docx]

**TITLE:** Differential Expression Profile of NLRs and AIM2 in Glioma and Implications for NLRP12 in Glioblastoma

**AUTHOR LIST AND AFFILIATIONS:**

Nidhi Sharma^1^, Shivanjali Saxena^1^, Ishan Agrawal^1^, Shalini Singh^1^, Varsha Srinivasan^1^, S. Arvind^1^, ^2^Sridhar Epari, Sushmita Paul^1^, Sushmita Jha^1*^

^1^Department of Bioscience and Bioengineering, Indian Institute of Technology, Jodhpur, India

^2^Department of Pathology, Tata Memorial Hospital, Mumbai, Maharashtra, India

**^*^CORRESPONDING AUTHOR** Sushmita Jha, PhD, Associate Professor, Department of Bioscience and Bioengineering, Indian Institute of Technology Jodhpur, Karwar, Jodhpur, India -342037.
eMail: sushmitajha@iitj.ac.in


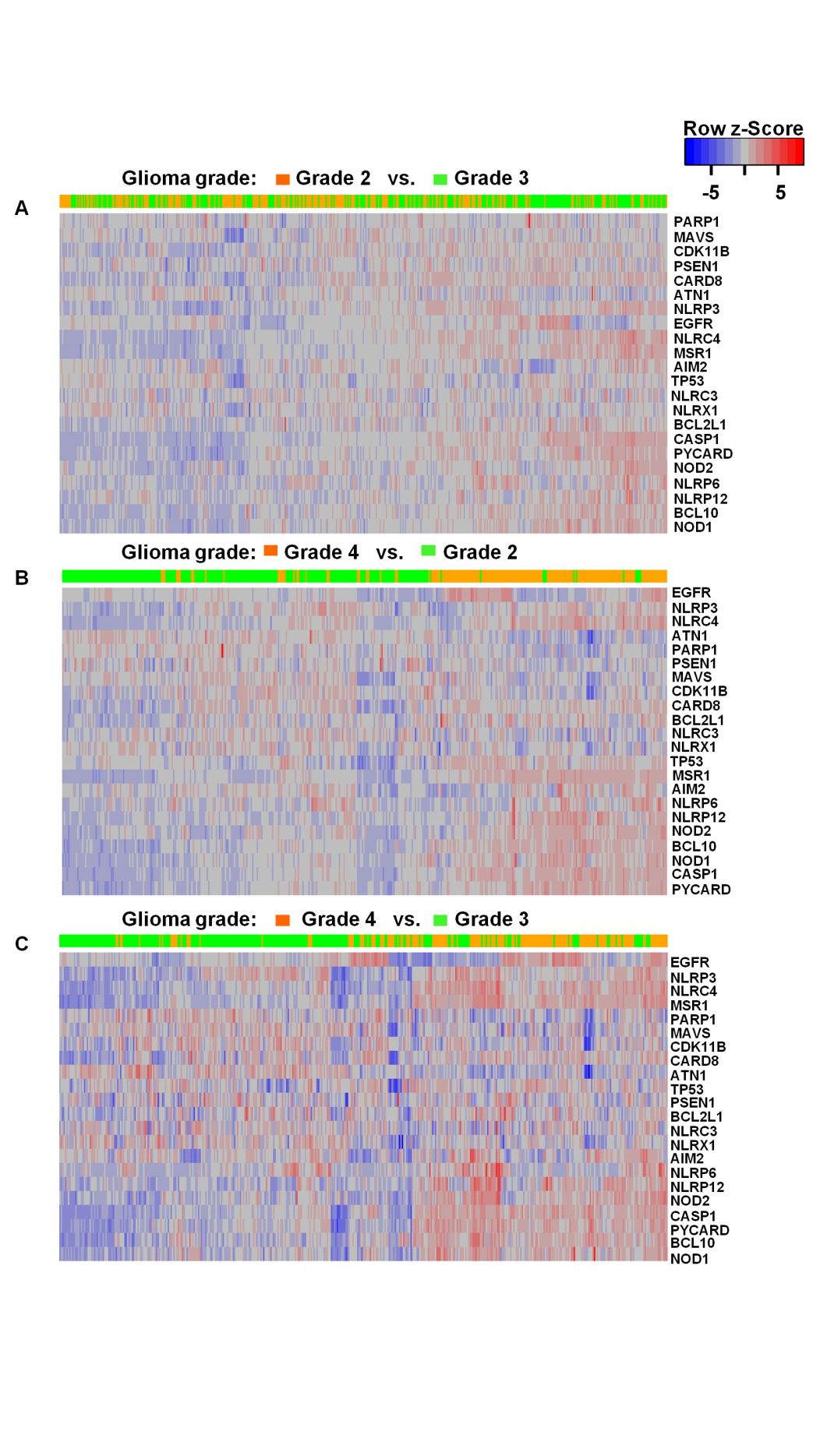


Supplementary Figure 1: *Heat map clustering representation for NLR gene expression in glioma.* (A) Shows NLR gene expression across the grade 2 (orange) - grade 3 (green) of LGG samples. (B, C) Shows characteristic NLR gene expression clusters between grade 4 (orange) – grade 2 (green) and grade 4 (orange) – grade 3 (green) glioma samples respectively. Here, relative up-regulated and down-regulated gene expression, are shown in red and blue respectively.

**
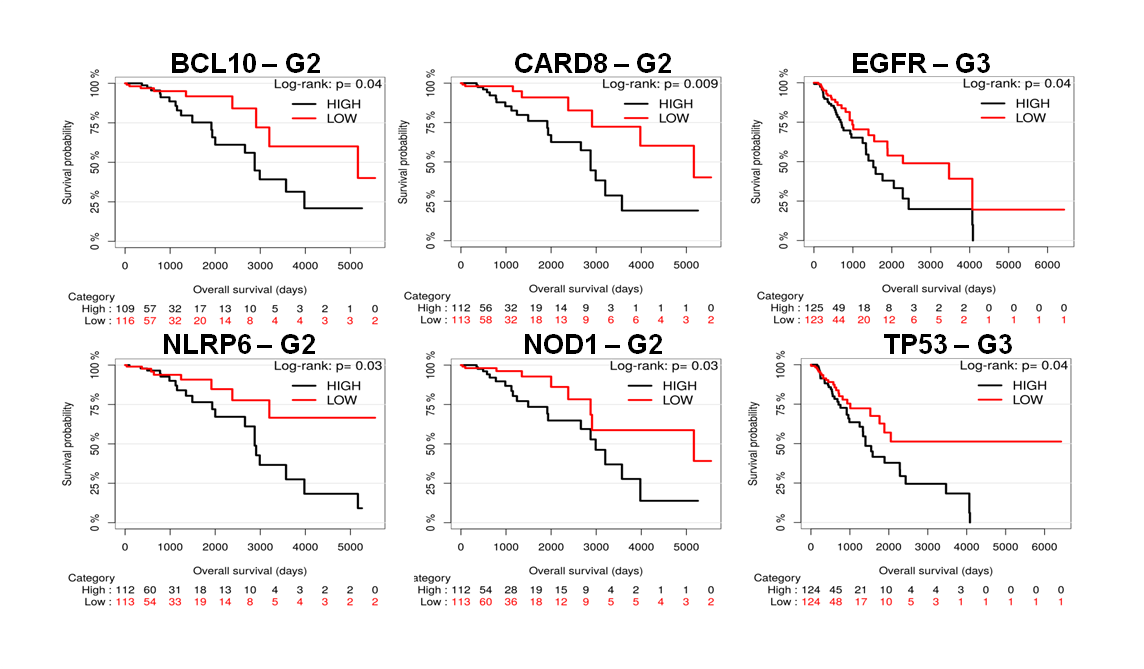
Supplementary Figure 2:** *Kaplan-Meier (KM) survival curves of low grade glioma and glioblastoma patients stratified by the expression levels of NLRs.* Shows genes having significant association with the glioma survival outcome in the LGG (grade 2, grade 3) patient samples.

**
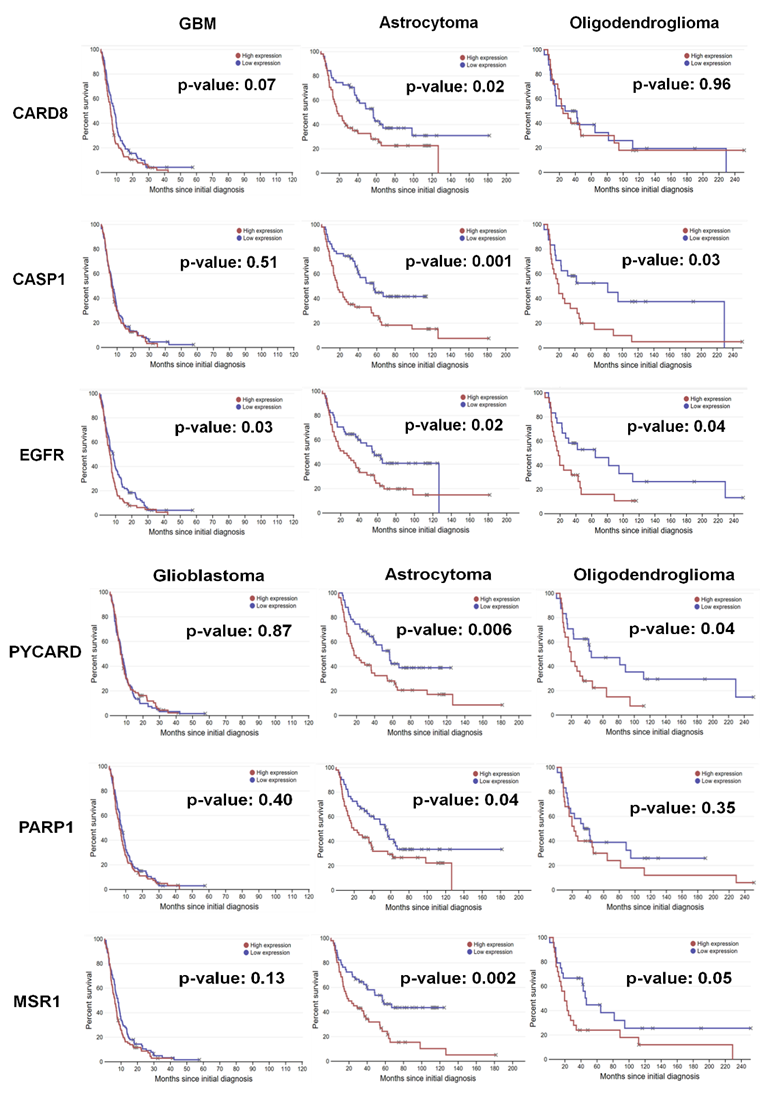
**

**Supplementary Figure 3:** *Kaplan-Meier (KM) survival curves of REMBRANDT GBM and LGG patients stratified by the expression levels of NLRs.* Here, in the REMBRANDT - LGG datasets, the LGG data has been analyzed based on two categories – astrocytoma and oligodendroglioma, provided by the revised molecular subtyping of GBM included in the WHO classification of CNS tumours ({Louis *et al.*, 2016). The figure shows the overall survival curve for the *CARD8, CASP1, EGFR, MSR1, PYCARD* and *PARP1* genes association with the glioma survival outcome.

**
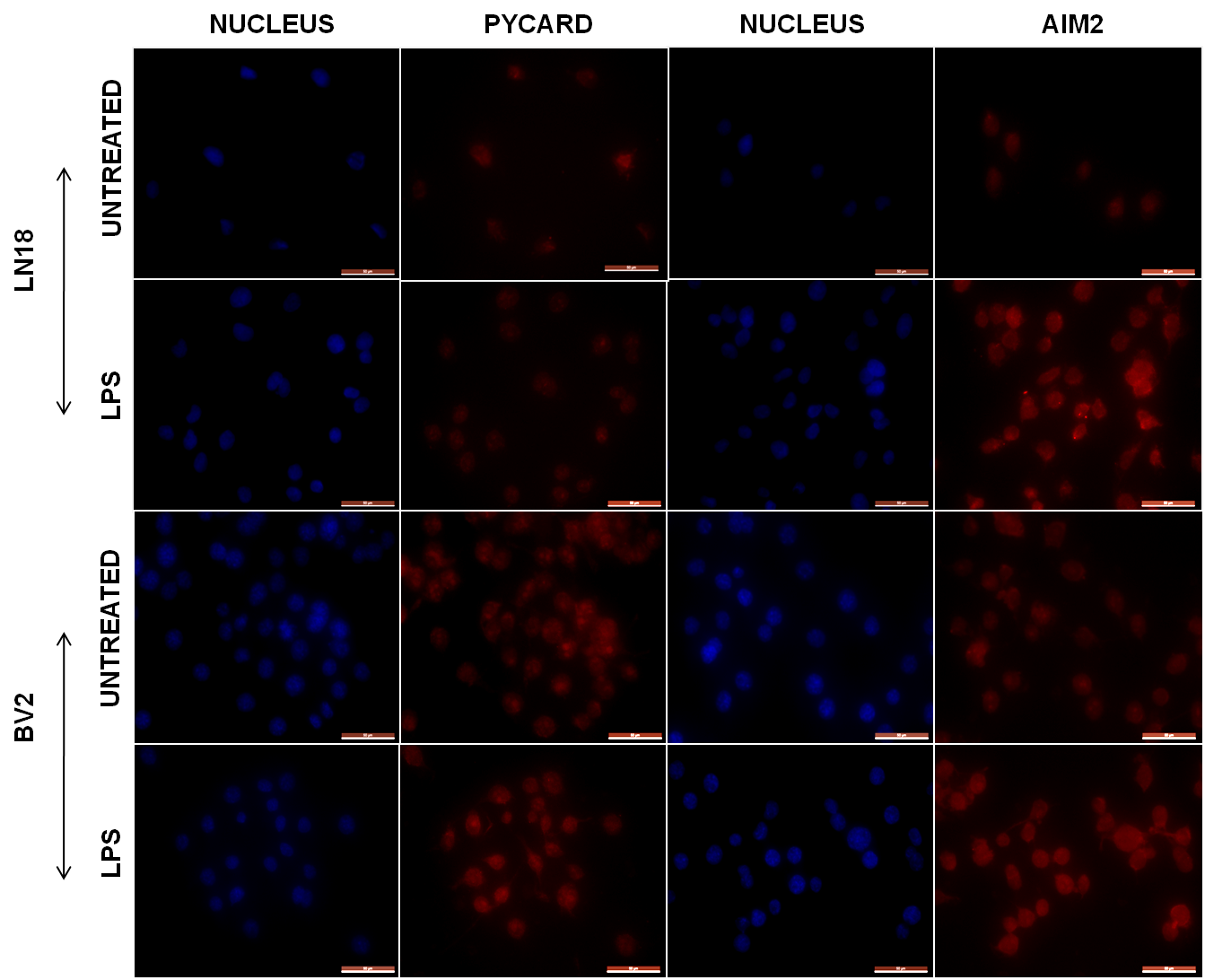
**

**Supplementary Figure 4:** *PYCARD and AIM2 expression in glioma and microglial cells.* **(a, b, c)** We have observed ASC/PYCARD and AIM2 protein expression in control and lipopolysaccharide-primed LN18 glioma and BV2 microglial cells. Nuclei were stained blue using DAPI. Magnification: 40X, Scale bar: 50μm**.**

**
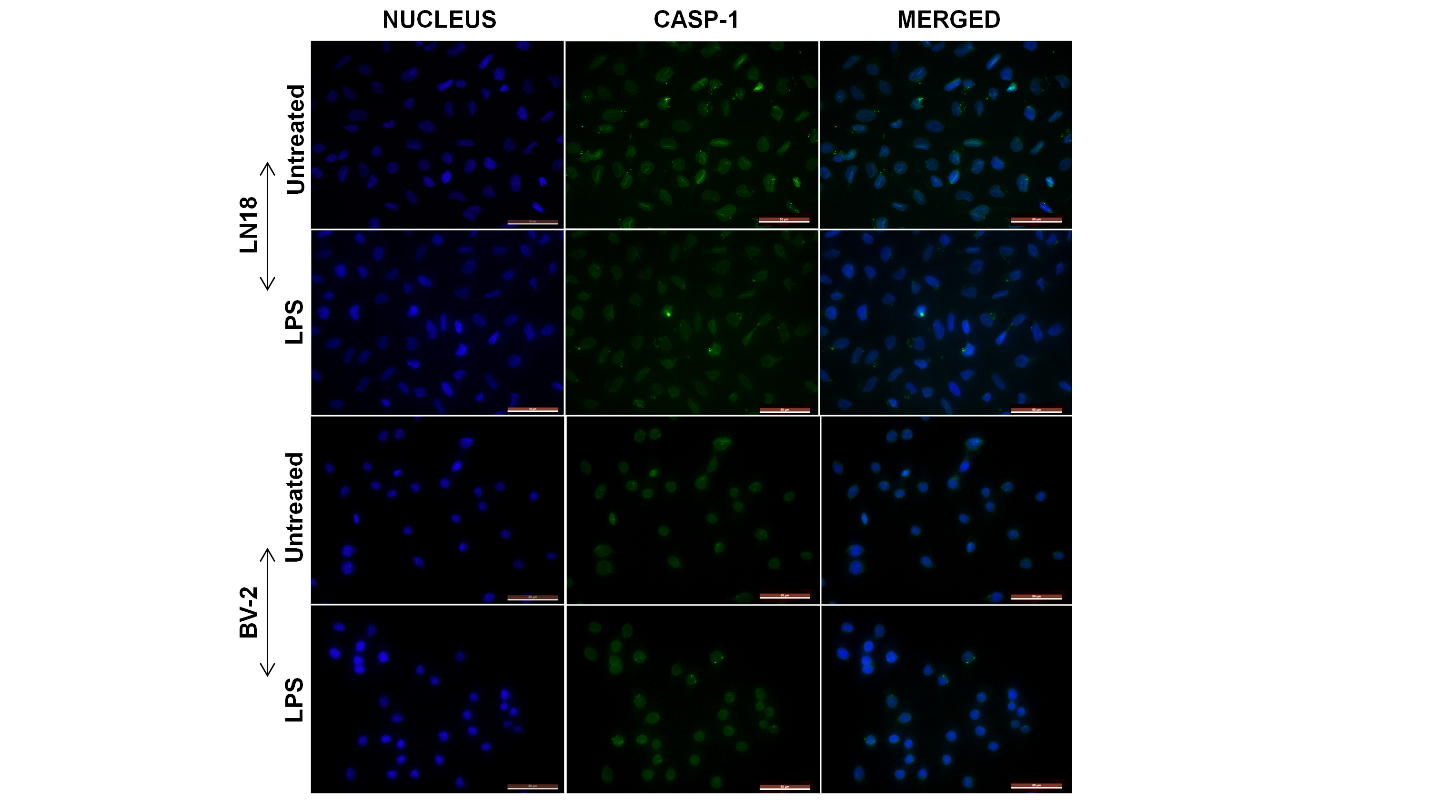
**

**Supplementary Figure 5:** *Caspase-1 expression in glioma and microglial cells.* **(a, b)** We have observed CASP-1 protein expression in control and lipopolysaccharide-primed LN18 glioma and BV2 microglial cells. Magnification: 40X, Scale bar: 50μm.


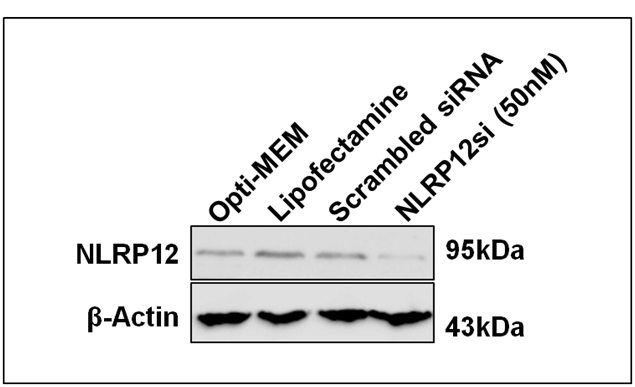


**Supplementary Figure 6:** NLRP12 knockdown in *NLRP12siRNA treated BV2 microglia cells.* The western blot shows decreased NLRP12 protein expression in NLRP12 siRNA (50nM) treated BV2 miroglia cells as compared to the control. Here, we have used Opti-MEM, Lipofectamine and scrambled siRNA (50nM) treated cells as controls. We found two NLRP12 (1:3000, GeneTex) bands, one at 95 kDa and other at 58 kDa (not shown). β-actin (1:5000, Santa Cruz) was taken as protein loading control. Anti-rabbit secondary antibody (1:10,000; Cell Signaling) and Anti-mouse (1:5000; Cell Signaling) were used against NLRP12 and β-Actin respectively.


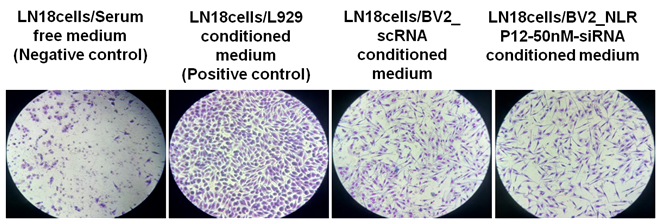


(c)


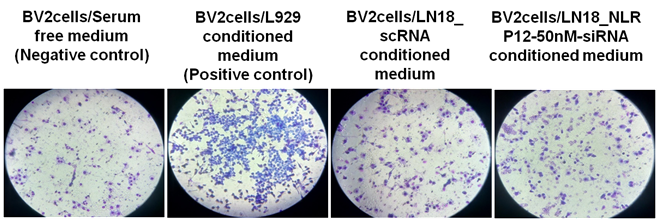


(a)

(b)


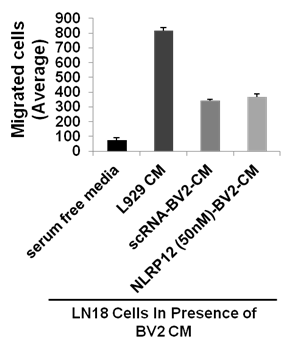


**Supplementary Figure 7:** *Effect of NLRP12 knockdown-CM on BV2 and LN18 cells*. (a) We incubated BV2 cells with NLRP12 siRNA (50nM) treated LN18 cell conditioned medium (CM) and observed the migration capacity of BV2 glioma cells in presence of LN18 (NLRP12 knockdown) - CM. (b) Similarly, we incubated LN18 cells with NLRP12 siRNA (50nM) treated BV2-CM and observed the migration capacity of LN18 glioma cells in presence of BV2 cell conditioned medium. (c) The effect of BV2 (NLRP12 siRNA treated) cell conditioned medium (CM) on LN18 cell migration was assessed using migration assay. 8 image sections per sample were quantifed for migration analysis. One- way ANOVA was performed to find the statistical significance of migration assay (*p-value < 0.05). The error bar indicates standard error of mean.

**
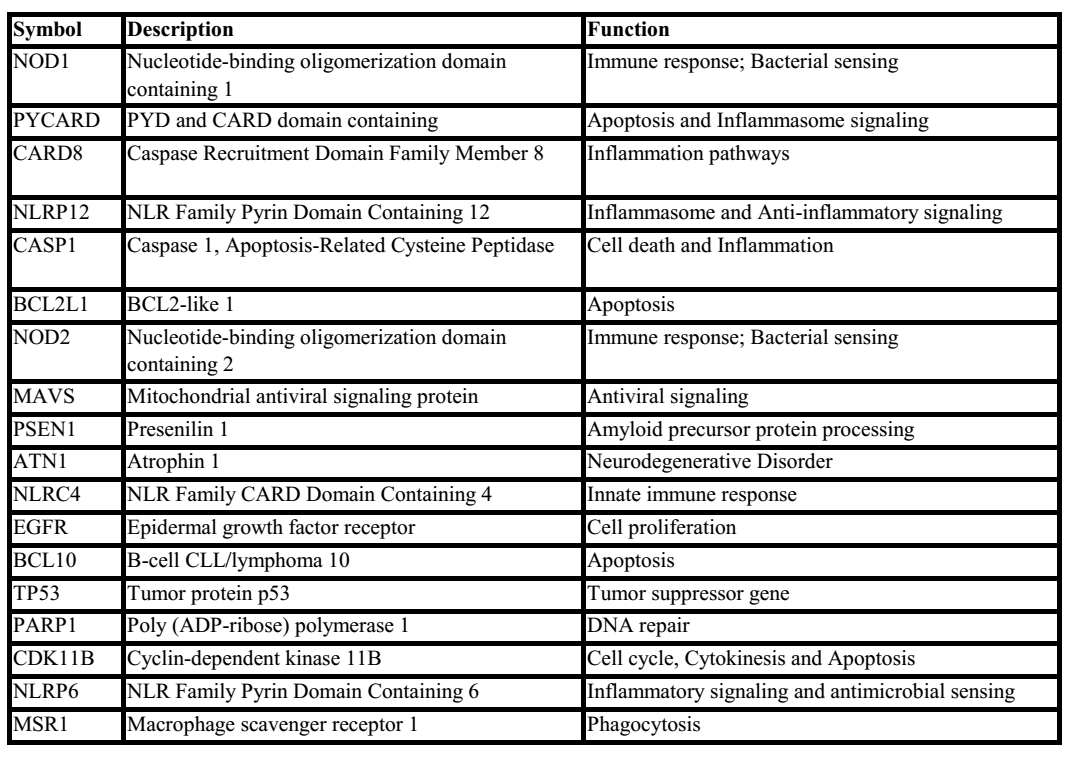
**

**Supplementary Table 1:**  *Description of differentially expressed genes in glioblastoma*

**
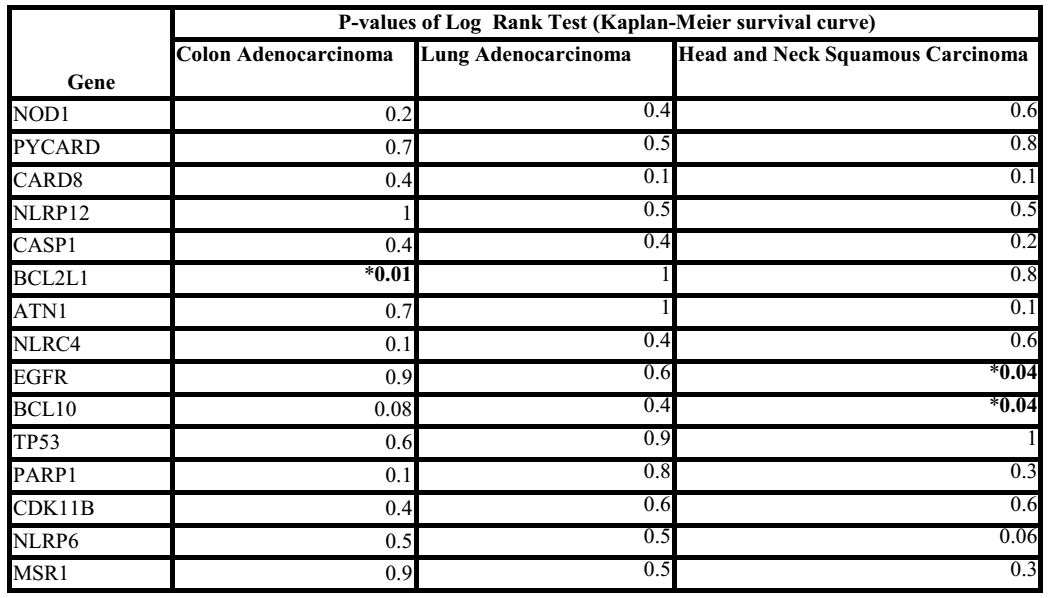
**

**Supplementary Table 2:** *P-values for Kaplan-Meier survival curve analysis of multiple TCGA cancer datasets.* *p-value <0.05 (log-rank test) is significant. Kaplan-Meier survival curves of pan-cancer analysis across TCGA – Colon adenocarcinoma, Lung adenocarcinoma and, Head and neck squamous cell carcinoma patients stratified by the expression levels of NLRs and NLR-associated genes.
